# Association of metformin administration with the serum levels of zinc and homocysteine in patients with type 2 diabetes: a cross-sectional study

**DOI:** 10.1007/s13340-025-00798-x

**Published:** 2025-02-08

**Authors:** Sadako Matsui, Chika Hiraishi, Ryo Sato, Takai Kojima, Keiichiro Matoba, Kei Fujimoto, Hiroshi Yoshida

**Affiliations:** 1 Food and Nutrition, Faculty of Human Sciences and design, Japan Women’s University, 2-8-1, Mejirodai, Bunkyo, Tokyo 112-8681 Japan; 2 Department of General Medicine, The Jikei University Kashiwa Hospital, 163-1, Kashiwashita, Kashiwa, Chiba 277-8567 Japan; 3 Department of Laboratory Medicine, The Jikei University Kashiwa Hospital, 163-1, Kashiwashita, Kashiwa, Chiba 277-8567 Japan; 4 Division of Diabetes, Metabolism and Endocrinology, The Jikei University Kashiwa Hospital, 163-1, Kashiwashita, Kashiwa, Chiba 277-8567 Japan; 5 Division of Diabetes, Metabolism and Endocrinology, The Jikei University Daisan Hospital, 4-11-1, Izumihoncho, Komae, Tokyo 201-8601 Japan; 6 Section of Internal Medicine of Metabolism and Nutrition, The Jikei University Graduate School of Medicine, 3-25-8, Nishishinbashi, Minato, Tokyo 105-8461 Japan

**Keywords:** Metformin, Zinc, Vitamin B12, Homocysteine, Diabetes kidney disease

## Abstract

**Background:**

Metformin treatment has a risk factor of reduced serum concentrations of vitamin B12 and zinc, indicating its association with homocysteine metabolism. However, this association remains to be clarified in patients with type 2 diabetes (T2DM) accompanied by kidney dysfunction.

**Methods:**

This cross-sectional study was conducted in 149 patients with T2DM (96 men, 53 women), including diabetic kidney disease. Serum concentrations of homocysteine, as well as vitamin B12, folic acid, and zinc, were measured in outpatient T2DM patients. The study subjects were divided into two groups: patients with and without metformin administration (Met [ +], n = 62; Met [ −], n = 87). To explore the effect of kidney function, we also analyzed the data after dividing all the patients according to kidney function (chronic kidney disease [CKD] group, n = 66; non-CKD group, n = 83).

**Results:**

The Met ( +) group exhibited significantly higher serum zinc levels and lower serum homocysteine levels than the Met ( −) group. In the non-CKD group, metformin administration was positively associated with serum zinc levels, as demonstrated by multiple linear regression analysis adjusted for confounding factors (*β* = 0.287, *p* = 0.021). However, no significant association between metformin administration and serum zinc levels was observed in the CKD group. Moreover, there were no associations between serum homocysteine levels and metformin administration.

**Conclusions:**

The relationship between metformin treatment and serum zinc levels differed based on the presence or absence of CKD in patients with T2DM.

## Introduction

Metformin provides a modest effect for weight loss and prevents macroangiopathy such as cardiovascular outcomes in patients with type 2 diabetes with obesity and insulin resistance [1, 2]. Based on therapeutic and patient-side clinical factors including comorbidities and complications, metformin therapy and comprehensive lifestyle modifications are generally recommended [3–5]. However, metformin therapy can cause taste disorders resulting from zinc deficiency; its chelating action results in binding to systemic zinc and increasing urinary zinc excretion, thereby leading to zinc deficiency (hypozincemia) [6]. Increased urinary zinc excretion and hypozincemia are often found in patients with diabetes and were reported to be significantly associated with glycated hemoglobin and insulin resistance [7]. Zinc deficiency is related to cardiovascular disease (CVD) risk, which includes hypertension, dyslipidemia, inflammation, and oxidative stress, and is reportedly associated with CVD events in patients with chronic kidney disease (CKD) [8]. Hypozincemia has been observed in patients with diabetic kidney disease (DKD) who are prone to serum zinc deficiency caused by increased urinary zinc excretion because of a kidney tubular disorder, and serum zinc concentrations are reportedly lower in patients with macroalbuminuria than in those with microalbuminuria [9]. Zinc functions as a coenzyme in homocysteine metabolism [10], and intestinal conjugase responsible for folate absorption is a zinc-dependent enzyme [11]. Moreover, kidney failure exhibits hyperhomocysteinemia, one of the nonclassical CVD risk factors [12–14]. Recently, serum zinc concentrations in patients with diabetes with normal kidney function were reported to be significantly higher in metformin users than in nonusers [15]. In addition, we reported that kidney function significantly influences serum homocysteine, accompanied by the concurrent relevance of nutritional factors (vitamin B12, folic acid, and zinc), in type 2 diabetes patients including DKD [16]. However, the relationship between metformin treatment and serum zinc concentrations remains poorly studied in patients with type 2 diabetes with CKD, who are more prone to hypozincemia and the degrees of metformin’s impacts on serum zinc also remain undefined.

Vitamin B12 serum concentrations in patients with type 2 diabetes receiving long-term metformin therapy are reduced by 14%–30%, of which 30% being vitamin B12 deficient [17]. Metformin treatment carries a risk of decreased serum vitamin B12 concentrations [17–23]. In this regard, the American Diabetes Association (ADA) guidelines recommend that physicians should monitor for potential vitamin B12 deficiency in patients with type 2 diabetes [18]. Moreover, the influence of metformin treatment on serum concentrations of vitamin B12, homocysteine, and folic acid or zinc in Japanese patients with type 2 diabetes on normal kidney function has been reported [15, 24, 25]. Thus, metformin therapy may influence the serum levels of zinc and vitamin B12 and may be related to homocysteine metabolism; however, this finding is still unclear in patients with type 2 diabetes including kidney dysfunction.

Therefore, this study aimed to explore the association between metformin therapy and the serum levels of zinc or homocysteine in patients with type 2 diabetes including DKD.

## Materials and methods

### Study design and population

**Fig. 1:**
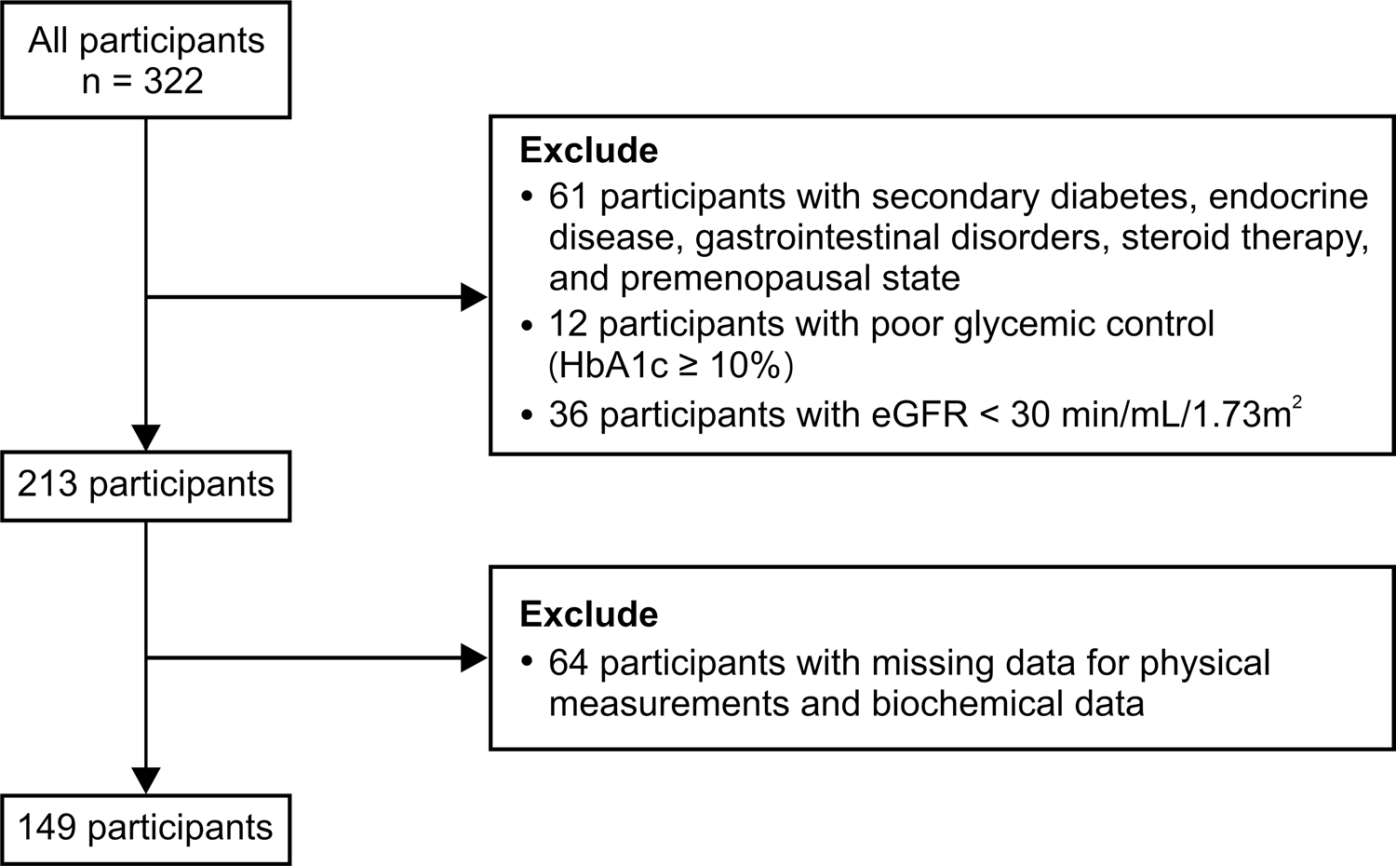
Flowchart depicting study participant enrollment

This cross-sectional study enrolled patients with type 2 diabetes who visited the outpatient clinic of the Division of Diabetes, Metabolism, and Endocrinology of the Internal Medicine department at the Jikei University Kashiwa Hospital between August 2018 and May 2020. We excluded patients with poor glycemic control [hemoglobin A1c (HbA1c) levels ≥ 10.0%], secondary diabetes, endocrine diseases, gastrointestinal disorders, steroid therapy, estimated glomerular filtration rate (eGFR) under 30 mL/min/1.73 m^2^, and missing data needed for this study. Finally, 149 patients (96 males and 53 females) participated in this study (Fig. 1).

The study was conducted in accordance with the Declaration of Helsinki and adhered to ethical guidelines for medical and health research involving human subjects. The protocol was approved by the Ethics Committee of the Jikei University School of Medicine (#30–010).

### Data collection and materials

The patients’ characteristics (age, sex, body mass index [BMI], blood pressure, medication status, alcohol and smoking habits, and histories of macrovascular disease) and laboratory data, including glucose, lipids, and uric acid (UA) levels in fasting samples, were collected from electronic medical records. Regular drinkers were those who habitually drank more than 60 g of alcohol/week, while current smokers were those who smoked daily and occasionally for more than 6 months and over the last 1 month. Macrovascular diseases included cerebral vascular disease, carotid artery disease, CVD, peripheral artery disease, and thoracic/abdominal aortic disease. The participants’ metformin doses (measured in mg per day) were recorded on the day of examination. Serum levels of total cholesterol (TC) and high-density lipoprotein cholesterol (HDL-C) were measured by direct methods, and low-density lipoprotein cholesterol (LDL-C) level was determined using Friedewald’s formula [26]. Furthermore, HbA1c was measured by high-performance liquid chromatography (glycohemoglobin analyzer HA-8190V). Creatinine and UA levels were measured by routine enzymatic methods (L-type Wako CRE M and L-type Wako U M, respectively). Serum levels of vitamin B12, folic acid, zinc, total homocysteine, and urinary albumin excretion (urinary albumin-to-creatinine ratio [UACR; mg/gCr]) were additionally measured using residual samples after confirmation by attending physicians when any of these was missing in laboratory data. As for the serum levels of folic acid and vitamin B12, we used chemiluminescent enzyme immunoassays (CLEIAs) (access folic acid and access B12, respectively). Moreover, the serum levels of zinc and total homocysteine were measured by colorimetry assay (espa Zn II) and high-performance liquid chromatography (YMC-Triart C18), respectively, and urinary albumin by immunonephelometry (Autowako microalbumin nc-x). In addition, eGFR (mL/min/1.73 m^2^) was calculated using the glomerular filtration rate estimation formula [27].

### Statistical analysis

To investigate the relationship between metformin administration and the various endpoints, we divided the patients into two groups: those who did not receive metformin (Met [ −] group, n = 87) and those who received metformin (Met [ +] group, n = 62). We compared the Met ( −) and Met ( +) groups using an unpaired t test for normally distributed variables and the Mann–Whitney *U* test for non-normally distributed variables. For categorical data, we used the Chi-square test.

Additionally, to explore the relationship between metformin administration and serum zinc or serum homocysteine levels, we performed multiple linear regression analyses (with forced entry), adjusting for confounding factors such as age, sex, BMI, eGFR, HbA1c, macrovascular disease (Model 1), and metformin administration (Model 2). We applied a logarithmic transformation for factors that were not normally distributed. To further investigate the impact of kidney function, we divided all patients into two groups: CKD, which included 66 patients, and non-CKD, comprising 83 patients. Next, we assessed the relationship between serum homocysteine levels and metformin administration or nutritional factors related to homocysteine. In our analysis, the dependent variable was the logarithm of homocysteine levels, while the independent variables included the serum concentrations of homocysteine-related nutritional factors: the logarithms of vitamin B12, folic acid, and zinc adjusting for confounding factors (Model 1) and metformin administration (Model 2). This analysis was performed using multiple linear regression.

Data are summarized using mean ± standard division (SD) or median and interquartile range for continuous variables, whereas frequencies and percentages are used for categorical variables. A two-sided *p* value < 0.05 was considered statistically significant for all analyses. Statistical analyses were conducted with SPSS Statistics 28 version 28.0.0.0 (IBM, Japan).

The total sample size was calculated to be 139, assuming an alpha level of 0.05, 80% power, a 0.15 effect size, and 15 predictors for the independent variable in a multiple regression analysis. This calculation was performed using G*Power version 3.1.9.7 (Franz Faul, Kiel University, Germany) [28].

## Results

### Participants’ characteristics

Table 1 shows the participants’ characteristics. The age, BMI, HbA1c, and eGFR were 68 ± 11 years, 25.8 ± 4.2  kg/m^2^, 7.6 ± 1.0%, and  61.4 ± 19.4 mL/min/1.73 m^2^, respectively. In all patients, 6 (4.0%) and 74 (49.7%) participants exhibited hypozincemia (< 60 µg/dL) and latent zinc deficiency (≥ 60 and < 80 µg/dL), respectively [6], and 16 (10.7%) demonstrated low serum levels of vitamin B12 (< 180 pmol/L).

**Table 1:** Overall characteristics of the participants and comparison between patients with and without metformin

Variables	Overall	Without metformin	With metformin	*p* value
Number (male / female) *	149 (96/53)	87 (59/28)	62 (37/25)	0.386
Age (years)	68 ± 11	72± 10	61 ± 10	< 0.001
BMI (kg/m^2^)	25.8 ± 4.2	25.3 ± 4.1	26.6 ± 4.3	0.051
Systolic blood pressure (mmHg)	131 ± 16	130 ± 16	131 ± 16	0.641
Diastolic blood pressure (mmHg)	74 ± 12	73 ± 10	75 ± 14	0.183
Albumin (g/dL)	4.0 ± 0.4	3.9 ± 0.4	4.2 ± 0.3	< 0.001
Hemoglobin (g/dL)	14.1± 1.6	13.8 ± 1.5	14.5 ± 1.6	0.011
Hematocrit (%)	41.7 ± 4.3	41.0 ± 4.3	42.6 ± 4.1	0.022
Creatinine (mg/dL)	0.96 ± 0.33	1.08 ± 0.35	0.79 ± 0.21	< 0.001
eGFR (mL/min/1.73 m^2^)	61.4 ± 19.4	53.1 ± 17.7	73.0 ± 15.5	< 0.001
Uric acid (mg/dL)	5.6 ± 1.4	5.8 ± 1.4	5.5 ± 1.3	0.167
UACR (mg/gCr) ^#^	53.1 (13.2, 234.5)	99.2 (17.8, 721.0)	25.9 (10.1, 86.7)	0.001
TC (mg/dL)	187 ± 37	190 ± 37	183 ± 38	0.307
HDL-C (mg/dL)	57 ± 15	60 ± 16	54 ± 12	0.010
LDL-C (mg/dL)	100 ± 31	101 ± 31	99 ± 30	0.691
TG (mg/dL) ^#^	128 (88, 182)	127 (87, 177)	131 (89, 197)	0.596
HbA1c (%)	7.6 ± 1.0	7.6 ± 1.1	7.4 ± 0.8	0.130
Homocysteine (nmol/mL) ^#^	10.7 (8.3, 13.4)	11.8 (9.2, 14.4)	9.2 (7.5, 11.4)	< 0.001
Vitamin B12 (pmol/L) ^#^	312 (230, 461)	342 (244, 527)	293 (227, 406)	0.117
Folic acid (µg/dL) ^#^	6.6 (5.2, 10.2)	7.2 (5.2, 10.8)	6.5 (4.8, 9.2)	0.342
Zinc (µg/dL)	80 ± 13	76 ± 12	86 ± 12	< 0.001
Metformin dosage (mg/day)	0 (0, 1,000)	N/A	1,000 (500, 1,000)	N/A

Data are expressed as number for the categorical variables or mean ± standard division and median (25^th^, 75^th^ percentile) for the contimuous variables

Unpaired *t* test or Mann–Whitney *U* test (#) for the statistical analysis of continuous variables

Chi-square test (*) for the statistical analysis of qualitative variables

A two-sided* p* value < 0.05 was considered statistically significant

*BMI* body mass index; *eGFR* estimated glomerular filtration rate; *UACR* urinary albumin-to-creatinine ratio; *HbA1c* hemoglobin A1c; *HDL-C* high-density lipoprotein cholesterol; *LDL-C* low-density lipoprotein cholesterol; *N/A* not applicable

We found 66 (44.3%) patients who met the DKD criteria. The age, BMI, HbA1c, and eGFR were 71 ± 10 years, 25.8 ± 3.9 kg/m^2^, 7.7 ± 1.1%, and 43.2 ± 8.5 mL/min/1.73 m^2^in the CKD group, whereas those in the non-CKD group were 65 ± 11 years, 25.9 ± 4.5 kg/m^2^, 7.4 ± 0.9%, 75.8 ± 12.2 mL/min/1.73 m^2^, respectively. Additionally, the proportion of metformin users was significantly lower in the CKD group than the non-CKD group, at 18.2% versus 60.2% (p < 0.001).

### Comparison of parameters in patients with and without metformin

Table 1 shows that the Met ( −) group was significantly older and had significantly lower values for albumin, hemoglobin, hematocrit, and eGFR than the Met ( +) group. Meanwhile, creatinine, UACR, HDL-C, and homocysteine levels were significantly higher in the Met ( −) group than in the Met ( +) group. Serum zinc concentration was significantly higher in the Met ( +) group at 86± 12 µg/dL compared to 76 ± 12 µg/dL in the Met ( −) group (*p* < 0.001). Vitamin B12 levels were slightly lower in the Met ( +) group (*p* = 0.117). Table 2 shows the participants’ drinking and smoking habits, macrovascular disease history, and medication status. Macrovascular disease history was significantly more observed in the Met ( −) group than in the other group (*p* = 0.019).

**Table 2:** Drinking and smoking habits, macrovascular disease history, and medication status

		Without metformin (*n* = 87)	With metformin (*n* = 62)	*p* value
Current smoker	Yes No	13 (14.9) 74 (85.1)	15 (24.2) 47 (75.8)	0.202
Regular drinker	Yes No	15 (17.2) 72 (82.3)	9 (14.5) 53 (85.5)	0.822
Macrovascular disease	Yes No	33 (37.9) 54 (62.1)	12 (19.4) 50 (80.6)	0.019
Antidiabetic drugs	User Nonuser	85 (97.7) 2 (2.3)	62 (100.0) 0 (0.0)	0.511
Antihypertensive drugs	User Nonuser	64 (73.6) 23 (26.4)	36 (58.1) 26 (41.9)	0.053
Lipid-lowering drugs	User Nonuser	50 (57.5) 37 (42.5)	35 (56.5) 27 (43.5)	1.000
Uric acid-lowering drugs	User Nonuser	21 (24.1) 66 (75.9)	7 (11.3) 55 (88.7)	0.057

Data are expressed as number (%)

Current smokers are defined as daily and occasional smokers who had smoked for more than 6 months and over the last 1 month

Regular drinkers are defined as those who were habitually drinking more than 60 g of alcohol per week

Macrovascular disease is defined as the presence of any of the following medical histories: cerebral vascular disease, carotid artery disease, cardiovascular disease, peripheral artery disease, and thoracic/abdominal aortic disease. A two-sided* p* value < 0.05 was considered statistically significant.

### Associations between metformin administration and the serum zinc level in all participants

Table 3 presents the results of the multiple linear regression analysis, with serum zinc concentrations as the dependent variables and metformin administration as the independent variables, after adjustment for confounding factors (age, sex, and BMI), eGFR, HbA1c, and macrovascular disease. Consequently, age, eGFR, and the use of metformin were independent factors influencing serum zinc concentration.

**Table 3:** Multiple linear regression analysis to identify factors associated with serum zinc levels in all participants

Variables		Overall (*n* = 149)	
		*β*	*p*-value
Model 1	Age	− 0.261	0.002
	Sex	0.078	0.315
	BMI	− 0.118	0.138
	eGFR	0.300	< 0.001
	HbA1c	0.068	0.373
	Macrovascular disease	0.081	0.305
Model 2	Age	− 0.193	0.029
	Sex	0.080	0.296
	BMI	− 0.130	0.097
	eGFR	0.215	0.016
	HbA1c	0.075	0.320
	Macrovascular disease	0.094	0.228
	Metformin administration	0.218	0.020

Data are expressed as standardized partial regression coefficient (*β*)

Sex is represented by male (= 1) or female (= 2), and metformin administration is represented by with (= 1) or without (= 0)

Macrovascular disease is represented by its presence (= 1) or absence (= 0) in medical histories

A two-sided* p* value < 0.05 was considered statistically significant. *BMI* body mass index; *CKD* chronic kidney disease; *eGFR* estimated glomerular filtration rate; *HbA1c* hemoglobin A1c

### Associations between metformin administration and serum zinc levels by kidney function

To explore associations between serum zinc levels and metformin administration on kidney function, we separately examined non-CKD and CKD groups. In the non-CKD group, metformin administration was an independent predictor of serum zinc levels (*β* = 0.287; *p* = 0.021); however, this association was not seen in the CKD group (Table 4).

**Table 4:** Multiple linear regression analysis for factors associated with serum zinc level in the non-CKD and CKD groups

Variables	non-CKD (*n* = 83)		CKD (*n* = 66)	
	*β*	*p*-value	*β*	*p*-value
Age	− 0.087	0.488	− 0.378	0.007
Sex	0.091	0.421	0.129	0.301
BMI	− 0.088	0.444	− 0.227	0.084
eGFR	0.082	0.462	0.253	0.086
HbA1c	0.094	0.393	0.131	0.320
Macrovascular disease	0.084	0.472	0.139	0.271
Metformin administration	0.287	0.021	0.032	0.831

Data are expressed as standardized partial regression coefficients (*β*)

Sex includes male (= 1) or female (= 2), and metformin administration includes with (= 1) or without (= 0)

Macrovascular disease is documented as present (= 1) or absent (= 0) in medical histories

A two-sided* p* value < 0.05 was considered statistically significant. *BMI* body mass index; *CKD* chronic kidney disease; *eGFR* estimated glomerular filtration rate; * HbA1c* hemoglobin A1c

### Associations of the serum homocysteine level and metformin administration in all participants

Table 5 shows that sex, eGFR, and log vitamin B12 and log folic acid were independent variables for serum homocysteine level with adjustment for confounders. However, serum homocysteine level showed no relationship with metformin administration.

**Table 5:** Multiple linear regression analysis for factors associated with serum homocysteine level in all participants

	Variables	β	*p* value
Model 1	Age	0.010	0.867
	Sex	− 0.306	< 0.001
	BMI	0.021	0.697
	eGFR	− 0.563	< 0.001
	HbA1c	0.015	0.764
	Macrovascular disease	− 0.055	0.306
	Log vitamin B12	− 0.249	< 0.001
	Log folic acid	− 0.296	< 0.001
	Zinc	− 0.057	0.310
Model 2	Age	− 0.007	0.904
	Sex	− 0.307	< 0.001
	BMI	0.026	0.628
	eGFR	− 0.541	< 0.001
	HbA1c	0.013	0.805
	Macrovascular disease	− 0.059	0.269
	Log vitamin B12	− 0.251	< 0.001
	Log folic acid	− 0.297	< 0.001
	Zinc	− 0.046	0.423
	Metformin administration	− 0.065	0.309

Data are expressed as standardized partial regression coefficient (*β*)

Sex is represented by male (= 1) or female (= 2), and metformin administration is represented by with (= 1) or without (= 0)

Macrovascular disease is represented by its presence (= 1) or absence (= 0) in medical histories

A two-sided* p* value < 0.05 was considered statistically significant

*BMI* body mass index; *eGFR* estimated glomerular filtration rate; *HbA1c* hemoglobin A1c; *Log vitamin B12* log-transformed vitamin B12; *Log folic acid  *log-transformed folic acid

## Discussion

We investigated the relationship between metformin treatment and serum zinc or homocysteine levels in patients with type 2 diabetes with DKD. The Met ( +) group had significantly higher serum zinc levels and lower serum homocysteine levels than the Met ( −) group. However, the differences in serum zinc or homocysteine concentrations between patients with the presence or absence of metformin administration in this study may have been strongly influenced by age, kidney function, and macroangiopathy. Furthermore, a recent study reported the declining tendency of serum zinc concentrations depending on the CKD stage in patients with diabetes [29]. Therefore, we examined whether metformin therapy could independently predict serum zinc levels and adjust influential factors using multiple linear regression analysis. Results suggested that the serum zinc concentration was associated with metformin therapy independent of glycemic control and kidney function only in the non-CKD group. Recently, Sakurai [15] et al. reported that in patients with diabetes with normal kidney function (eGFR ≥ 60 mL/min/1.73 m^2^), the serum zinc concentration was significantly higher in those treated with metformin than in their controls (without metformin administration), consistent with our present results; however, this finding was not seen in our patients with DKD.

Metformin is renally excreted, and its known adverse effects include gastrointestinal symptoms such as diarrhea and lactic acidosis often in elderly adults and patients with impaired kidney function. These patients must be treated carefully, so that they are not recommended to take metformin. Owing to the low number of patients in the CKD group receiving metformin, no correlation was found between serum zinc levels and metformin use in this group.

Conversely, metformin reportedly inhibits the activation of hypoxia-inducible factor-1α in human renal proximal tubular epithelial cells and protects against tubular injury by restoring chronic hypoxia in rat models with type 2 diabetes [30]. Some zinc transporters are expressed in the proximal tubules and might be involved in zinc reabsorption [31]. As such, metformin may preserve serum zinc concentrations via its actions in the kidney.

Further, we sought to determine the association between metformin therapy and the serum homocysteine level or the serum levels of nutritional factors related to homocysteine metabolism. The association between metformin use and low vitamin B12 levels in type 2 diabetes mellitus patients is well established, and serum levels of homocysteine and methylmalonic acid are evaluated in vitamin B12 deficiency and utilized in diagnosing cellular vitamin B12 deficiency [32]. Serum homocysteine levels are reduced after administration of vitamin B12 in metformin-treated patients with vitamin B12 deficiency [25]. Moreover, serum vitamin B12 deficiency and/or chronic kidney insufficiency may be the primary cause of the most elevated total homocysteine concentrations in elderly adults [33]. We observed the relevance of serum homocysteine and serum vitamin B12 on folic acid concentrations after adjusting for metformin and other confounding factors, suggesting that metformin administration has no significant effect on the relationship between serum homocysteine and vitamin B12. However, the serum levels of vitamin B12 and folic acid were higher in the Met ( −) group than the Met ( +) group, and serum homocysteine levels was lower than in the Met ( +) group. An enterohepatic circulation of vitamin B12 has been suggested to be a major role in conserving vitamin B12, because the amount of vitamin B12 excreted in the bile is much higher than that excreted in the urine. Feces and vitamin B12 are reabsorbed at the terminal ileum [34]; in these regards, the serum vitamin B12 concentration may not reflect vitamin B12 deficiency at the tissue or cellular level in the present study. Furthermore, an anionic inhibition of the membrane transport of 5-methyltetrahydrofolate occurs in patients with CKD with a decrease in the rate of intracellular folate incorporation [35]; thus, serum folic acid levels also might not reflect intracellular vitamin B12 deficiency.

Metformin-induced vitamin B12 deficiency was investigated among Western country patients with type 2 diabetes. Metformin doses were found to be approximately 5–6 times higher in those patients than in Japanese patients with type 2 diabetes; these differences might influence the results in respective studies. Vitamin B12 plays an important role in red blood cell formation and brain/nervous system function, and metformin treatment may worsen pernicious anemia and peripheral neuropathy in patients with type 2 diabetes, attributed to blood vitamin B12 deficiency [36]. Additionally, metformin has effects that extend beyond the pancreas. It enhances lipid metabolism, inhibits gluconeogenesis, and promotes glucose uptake in muscles. Among the lipid metabolism indices we examined, the concentration of HDL-C was significantly lower in the Met ( +) group. In contrast, the concentration of LDL-C was higher in the Met ( −) group. There were no differences in the use of lipid-lowering drugs between these groups, indicating that unknown factors may have influenced the variations in lipid metabolism data observed in our study.

This study has limitations as follows. It was a cross-sectional study conducted at a single institution, with a relatively small sample size; therefore, we could not elucidate the mechanism by which metformin exerts its action on serum zinc concentrations or assess the influences of diuretics, sodium-glucose cotransporter 2 inhibitors, and other drugs that act on the kidney tubules. In addition, we did not investigate patients’ dietary habits or history of supplementation using, for example, vitamin B12 and folate or the duration of metformin treatment. Given the role of zinc in oxidative stress and that the serum concentrations of vitamins and minerals may be influenced by the presence of disease complications and a variety of treatments [37, 38], monitoring the serum concentrations of vitamins and minerals would be necessary to prevent the development of complications in patients with type 2 diabetes.

## Conclusions

The present study suggested a positive association between metformin treatment and serum zinc levels after adjustment for confounders. Still, this association differed according to the presence or absence of CKD in patients with type 2 diabetes. Thus, achieving the favorable effect of metformin treatment on serum zinc levels may depend on kidney function. Meanwhile, metformin treatment showed no major effects on serum homocysteine levels, but relevant intervention studies may be needed.

## Data Availability

The datasets used and analyzed in the current study are available from the corresponding authors upon reasonable request.
